# A Health Census

**Published:** 1901-01-12

**Authors:** 


### The Hospital

A Journal of

The Medical Sciences and Hospital Administration.

Vol. XXIX.—No. 746.] Edited by Sir Henry Burdett, K.C.B., and Solomon C. Smith, M.D., M.R.C.P. [January 12, 1901.

### A Health Census.

The arrival every ten years of the period at which during the past century it has been customary again to number the people has always given occasion to anxious inquirers, of many sorts, to suggest how easily, and with how much advantage to the public, their own particular form of curiosity could be satisfied; but we do not remember ever to have seen it suggested that the opportunity should be employed for the purpose of ascertaining the actual prevalence of illness, or, in other words, the precise percentage of the community who on the census day are withdrawn from the active occupations of life by the effects of some form of disease. Certain kinds of permanent disablement, such as those produced by blindness or deaf-mutism, are now recorded, but we do not know of any figures to display the daily loss of national energy and working capability which are dependent upon the sick bed. Yet surely there are few conditions better worthy of being studied, from the statistical point of view, or better calculated, when properly handled, to impress upon all concerned the value of health as an Imperial asset, or as an element to be taken into account in any sound estimate of our powers, either of resistance or of attack. The most important part of such information would be that covering the age-period from five to fifteen, as the school time for a preponderating number of the community; and the ages from twenty-five to sixty, as those of complete industrial or professional activity. We should, no doubt, obtain a formidable aggregate of invalids if we were to take merely the total number of medical practitioners in the United Kingdom and to assume that each of them, in order to live, must have some modest daily average of persons requiring his assistance; but any such calculation would be vitiated, as regards its practical value, by the consideration that many of these invalids, if not most of them, would be already placed by age, sex, or other circumstances outside of the current of daily activity, insomuch that the visit of the doctor would be more correctly an indication of their ability to remunerate his attendance than an evidence that it had become to them a matter of necessity. What is wanted is to ascertain how many persons, otherwise or ordinarily busy and capable, are temporarily withdrawn from their customary pursuits and converted either into helpless burdens upon the community or into mere consumers of their own previously acquired resources —in other words, what is the daily loss of bodily or mental capacity inflicted upon the community by disease. The adage, "*Salus populi suprema lex,*" would acquire greatly increased force from some vivid presentment of the amount of the fine which we pay for disregarding it. The figures would bring home, even to the dullest imagination that ever wandered into the paths of politics, at least some faint conception of the money value of the public health, and of the importance of adequate measures for its preservation or protection.

The notification in the census return of the mere fact of permanent or (presumably) temporary disablement by illness would constitute but a very small increase to the information now required from the householder, and it probably would not be prudent to ask for more than this on the present occasion. It would, of course, be in addition to the description of the occupation which is now required, and in this way it would at once form a valuable element in that study of the diseases incidental to different forms of industry to which Dr. Tatham has devoted so much time and labour, and which, so far, has been conducted on the basis of the mortality returns alone. Even if carried only to this extent, nothing is more probable than that the census of 1911 would show a diminution in the number of the sick as compared with 1901. But, in the United States, it is found practicable to include within the census inquiry a large number of particulars of which, in the United Kingdom, we take no note, and, if a return of the amount of disablement from sickness were once effected, it might be improved on future occasions by statements as to the causes of disablement, resting, of course, upon medical authority. Nothing, however, is born full-grown, and the elimination of ineffectives from the figures standing to the credit of the nation would be an essential preliminary to any investigation of the conditions by which the ineffectiveness had been produced. The question whether a man be fit or unfit, sick or well, is of at least as much importance as the question whether he be thirty-five or forty, and admits of being answered with equal simplicity and directness. The question of causes might sometimes involve other considerations, and might not improbably become a stimulus to inveracity. The attempt might, nevertheless, be made, and only experience could show to what extent the replies were likely to be trustworthy. We cannot but think that a "Health Census," even in an early and tentative form, would afford ample justification for those by whom it was advocated, and would form a basis for future and more extended inquiries, the results of which would be of great national utility.

